# Implementation and first-year screening results of an ocular telehealth system for diabetic retinopathy in China

**DOI:** 10.1186/1472-6963-11-250

**Published:** 2011-10-04

**Authors:** Jinjuan Peng, Haidong Zou, Weiwei Wang, Jiong Fu, Bingjie Shen, Xuelin Bai, Xun Xu, Xi Zhang

**Affiliations:** 1 Department of Ophthalmology, Shanghai First People's Hospital, affiliated Shanghai Jiaotong University, Shanghai 200080, China; 2 Beixinjing community health service center, Shanghai, China

## Abstract

**Background:**

To describe implementation and first-year screening results of the first Chinese telehealth system for diabetic retinopathy (DR) - the Beixinjing Community Diabetic Retinopathy Telehealth system (BCDRT).

**Methods:**

BCDRT implementation was based on the acquisition of adequate digital retinographs, secure digital transmission, storage and retrieval of participants' data and reader-generated medical reports. Local diabetic residents meeting inclusion criteria were enrolled into the BCDRT system beginning in 2009. Participants recommended for further in-person examination with ophthalmologists were followed, and the consistencies in diagnoses between BCDRT and ophthalmologists for DR or macular edema were calculated.

**Results:**

A total of 471 diabetic residents participated in BCDRT screening in 2009. The proportions of total DR, proliferative DR, and diabetic macular edema were 24.42% (115 patients), 2.12% (10 patients) and 6.47% (24 patients), respectively: 56 patients consulted ophthalmologists for further in-person retinal examination with funduscopy after pupil dilation. High rates of consistency between BCDRT screening and ophthalmologists were observed for macular edema (Kappa = 0.81), moderate or severe non-proliferative DR grade (Kappa = 0.92), and other DR grades (Kappa = 1). A total of 456 (96.82%) patients were willing to participate in the next BCDRT screening.

**Conclusions:**

BCDRT was a reliable and valid system for DR screening, and offers the potential to increase DR annual screening rates in local residents.

## Background

It is well recognized that final visual loss in diabetic retinopathy (DR) generally occurs after early stages that are chronic and asymptomatic. Therefore, 20 years ago, the World Health Organization recommended annual eye fundus examinations for all diabetic patients [1]. Until recently, however, the proportion of diabetic patients undergoing annual eye examinations was still low in developed countries; for example, 40%-50% in the U.S [2]. It may be intuitively concluded that the proportion in most underdeveloped countries, such as China, would be even less. Today, the Chinese population with diabetes is estimated to be 92.4 million in adults [3], and at least 20% of the diabetic population are likely to suffer from DR [4,5]. Therefore, the current number of Chinese diabetics without regular eye fundus examination is roughly estimated at more than 46 million (50% of 92.4 million), including at least 9 million(20% of 46 million) DR patients.

Currently, Early Treatment Diabetic Retinopathy Study (ETDRS) thirty-degree, stereo seven-standard fields, color, 35-mm slides are well-accepted as a "gold" standard for evaluating DR [6]. However, in China, ETDRS photography remains largely impractical for use in annual ophthalmic evaluations recommended for patients with diabetes, especially in primary care settings, because of several key disadvantages: requirements for skilled photographers and pupil dilation; the cost and inconvenience of film processing and archiving; the relatively long time spent on diagnosis. The traditional DR screening method in China relies on in-person funduscopic examination after pupil dilation by ophthalmologists. In the past 10 years, a screening method based on digital retinograph acquisition and telemedicine technology has been adopted in developed countries, in large part due to the high potential of this method for improving annual DR screening rates [2,7-12]. However, only a few studies address the DR telemedicine screening experience in underdeveloped countries such as India and Nepal [13,14]. As far as we know, the barriers to DR telemedicine system implementation in underdeveloped countries include relatively little knowledge about DR and poor follow-up compliance among the general diabetic population, poor ophthalmic infrastructure in primary care settings, and deficiency of wireless communication hardware and software to build specific local area networks for data transfer. Beginning in 2008, we began to establish the first ocular telehealth system for DR in China -the Beixinjing Community Diabetic Retinopathy Telehealth system (BCDRT). First, we heightened DR awareness along with the concept of BCDRT in the general population of the Beixinjing Community via a local newspaper, community broadcasting, community health science lecture and self-made promotional materials. Every diabetic patient presenting to local primary care settings, was informed about the construction and working procedures of BCDRT, with the realization that BCDRT would be accepted by most of them. Then, we trained personnel in the BCDRT system, including primary care physicians (PCPs), the engineer and ophthalmologists, transferring essential knowledge and skills. In a pilot study with 109 candidates, we confirmed the qualifications of trained personnel, and tested the image acquisition hardware, image analysis software, digital data transmission, and storage and retrieval systems for BCDRT [15]. In this report we present: (1) a detailed description of BCDRT development, (2) screening results of BCDRT in 2009, and (3) evidence that DR screening with BCDRT was as valid as traditional in-person screening.

## Methods

The objective of BCDRT is to serve diabetic residents living in the Beixinjing community of Shanghai (2.22 km2 and 40,477 inhabitants in 2009). BCDRT is comprised of the Reading Center located in the Shanghai First People's Hospital, the Peripheral Unit and the data server located in the Beixinjing community health service center (the sole primary care setting of the community).

Two PCPs in the Peripheral Unit, who had taken part in the pilot study [16], performed the following procedures: recording diabetic participants' personal information (gender, date of birth) and medical history (diagnosis and treatment of diabetes and eye diseases, along with the date of last eye examination (if available); confirming visual acuity with an ETDRS visual chart, and acquiring two 45-degree digital retinographs per eye without pupil dilation via a non-mydriatic funduscopic camera (CR-DGI, Canon, Tokyo, Japan). The realization method of retinographs was similar to the technique indicated in the EURODIAB study: one centering on the optic disk and the other on the macula [16]. The images were true color (24 bits) Joint Photograph Experts Group format at a resolution of 3504 × 2336 pixels, usually in 1175-1482 kb [15]. The PCPs viewed retinographs immediately, and repeated the acquisition process in cases of unsatisfactory image quality. Each of them was able to complete the procedures in one individual in 10-15 minutes. The process required approximately one-half day's work, and the two PCPs submitted the entire data from at least 40 participants, including retinographs in a standard memory card, to one licensed computer engineer in the data server.

The engineer immediately input the data into a self-made database, then transmitted the digital data to the Reading Center via internet. He guaranteed at least a 128 KB/sec internet connection speed and allowed a secure socket link connection at 128-bit encryption to verify the identity of users when setting up local user accounts. The engineer also ensured safe access to the data by assigning identification numbers and passwords to the users. All tasks carried out during data transmission and storage were registered in an auditing file, including the user name and identification number, date and time of the event, patient and identification number and examination number.

One ophthalmologist ("reader") in the Reading Center, with 12 years experience with retinal diseases, downloaded the data and read the images with a 17-inch true-color monitor (resolution: 1280 × 1024 pixels and 24-bit color; Trinitron Multiscan G500, Sony, Japan) by the end of the next business day. With commercial image processing software (Photoshop, Adobe, Seattle, U.S.), the images were enhanced by contrast, brightness and zoom controls to facilitate accurate analysis whenever necessary. The reader took 5-7 minutes per patient to read and generate a report that was later uploaded to the data server. The report included diagnosis of DR grade or macular edema according to the international DR classification [17], and identification of impaired visual acuity caused by non-diabetic ocular disorders, along with medical recommendations. Similar to some well-established telemedicine systems, such as DigiScope and OPHDIAT [7,9], the BCDRT protocol used nonstereo images, and it is not possible to detect macular thickening with this imaging modality. Therefore, the detection of macular edema is based on the surrogate lesion of hard exudates in the central macular field, or within one disc diameter of the center of the macula. The lesion is considered usually associated with adjacent retinal thickening and is predictive of the presence of macular edema [7,8]. On retrospectively analyzing the seven standard fields form and the ETDRS photographic data base, such criteria have a sensitivity of 94% for detection of clinically significant macular edema [18]. In case the existence of hard exudates in the central macular field is suspected, but cannot be confirmed, a visual acuity below 63 ETDRS letters (20/62.5) will trigger an automatic diagnosis of macular edema. Medical advice to the diabetic participants included mainly two recommendations: (1) consulting an experienced ophthalmologist for further in-person eye examination after pupil dilation, as soon as possible, in cases of moderate non-proliferative DR (NPDR), or worse [9], macular edema or ungradable photographs; or (2) attending another eye screening within the next year in case of normal appearance or mild NPDR. The criteria for ungradable images were the same in former reports: when the large vessels of the temporal arcades were blurred or more than one-third of the picture was blurred unless referable retinopathy was detected, an eye was considered ungradable [19]. When unexpected diseases were detected that required urgent attention, the reader promptly phoned the PCPs. The engineer in the data server downloaded the reader-generated reports, saved them into the database, and provided print-copies to the PCPs, who later informed the participants and scheduled the next screening time if accepted.

The construction of BCDRT was finished at the end of November 2008. After a one-month period of pilot work, we began to enroll diabetic residents into BCDRT screening from January 1, 2009. The inclusion criteria were set as: confirmed diabetic patients, without psychiatric disorders, who were willing to participate in BCDRT screening and who provided informed consent for this study.

We contacted the recommended participant for further in-person examination 1 month after his/her BCDRT examination, and acquired diagnoses from the ophthalmologists. Then, the consistencies between the telehealth system (BCDRT) and the ophthalmologists in regard to DR or diabetic macular edema diagnoses were calculated using Kappa value with SPSS V10.0 Statistical package (Chicago, IL). This study was conducted according to the tenets of the Declaration of Helsinki, and was approved by the Institutional Review Board at Shanghai First People's Hospital.

## Results

A total of 471 diabetic residents from the PCPs service participated in BCDRT screening in 2009. Their ages ranged from 19 to 89 years, with a mean (± standard deviation, SD) of 71.45 (± 6.93) years. All the residents were confirmed with type 2 diabetes mellitus, and the mean (± SD) duration of diabetes was 6.73(± 4.57) years (range: 0-35). Other personal characteristics are listed in Table 1. Only 101 (20.91%) residents had received a traditional in-person eye fundus examination within the past year; 456 (96.82%) residents were willing to participate in the next BCDRT screening. In 23 residents (4.88%), retinographs of at least one eye could not be analyzed, caused by media opacities, small pupil size, strabismus, nystagmus and head tremor. DR was detected in 115 residents, so the proportion of total DR was 24.42%. The patient numbers and proportions of different DR stages and diabetic macular edema are shown in Table 1.

**Table 1:** Screening results of BCDRT in 2009*

	N (%)
Total diabetic residents screened	471 (100)
Gender	
Male	181 (38.43)
Female	290 (61.57)
Age	
< 60 years old	159 (33.76)
> = 60 years old	312 (66.24)
Treatment of diabetes	
insulin or oral hypoglycemic agents	353 (74.95)
lifestyle intervention alone (diet, regular exercise, or both)	118 (25.05)
Previously diagnosed with DR	35 (7.43%)
Previous treatment of DR	
laser coaglulation	13 (2.76)
vitreoretinal surgery	2 (0.42)
Visual acuity of the better-seeing eye	
20/20 to 20/25	279 (59.24)
20/30 to 20/50	122 (25.90)
20/60 to 20/100	50 (10.62)
20/200 to 20/400	17 (3.61)
worse than 20/400	3 (0.64)
DR detected by BCDRT	
Total	115 (24.42)
Mild NPDR	71 (15.07)
Moderate NPDR	26 (5.52)
Severe NPDR	8 (1.70)
Proliferative DR	10 (2.12)
Diabetic macular edema detected by BCDRT	24 (6.47)

* BCDRT: the Beixinjing Community Diabetic Retinopathy Telehealth system; DR: diabetic retinopathy; NPDR: non proliferative diabetic retinopathy; PDR: proliferative diabetic retinopathy

A total of 56 patients were recommended for further in-person examination for DR or macular edema, and they consulted four ophthalmologists, all with more than 10 years experience in retinal diseases, during 1 month after BCDRT examination. Since the ophthalmologists all conducted retinal examination with funduscopy after pupil dilation, which is similar to the traditional Chinese DR screening method, we hypothesized that their diagnoses of DR or macular edema represented results from the traditional DR screening method. The ophthalmologists confirmed the BCDRT diagnoses of macular edema in 22 patients, and observed another 3 macular edema patients (Table 2). Thus, a high consistency was confirmed (Kappa = 0.81) between BCDRT screening and the traditional screening method for macular edema diagnosis. When considering moderate or severe NPDR grade diagnosis, the two screening methods were highly concordant in a total of 34 patients (Kappa = 0.92). The diagnostic conclusion of proliferative DR (PDR), or mild NPDR or less, was the same between the two screening methods (Kappa = 1) (Table 3). The ophthalmologists all agree with the various abnormalities of PDR in 10 patients. There were 10 patients diagnosed as PDR according to BCDRT diagnoses, 7 of whom were classified as preretinal or with vitreous hemorrhage, and the remaining 3 were classified as retinal neovascularization.

**Table 2:** Diagnoses of diabetic macular edema from BCDRT and ophthalmologists in 56 participants*

Diagnoses from BCDRT	Diagnoses from Ophthalmologists		
	
	macular edema	no macular edema	Total
macular edema	22	2	24
no macular edema	3	29	32
Total	25	31	56

* BCDRT: the Beixinjing Community Diabetic Retinopathy Telehealth system; DR: diabetic retinopathy; NPDR: non proliferative diabetic retinopathy; PDR: proliferative diabetic retinopathy

**Table 3:** Diagnoses of DR from BCDRT and ophthalmologists in 56 participants*

Diagnoses from BCDRT	Diagnoses from Ophthalmologists				
	
	Mild NPDR or less	Moderate NPDR	Severe NPDR	PDR	Total
Mild NPDR or less	12	0	0	0	12
Moderate NPDR	0	25	1	0	26
Severe NPDR	0	2	6	0	8
Proliferative DR	0	0	0	10	10
Total	12	27	7	10	44

* BCDRT: the Beixinjing Community Diabetic Retinopathy Telehealth system; DR: diabetic retinopathy; NPDR: non proliferative diabetic retinopathy; PDR: proliferative diabetic retinopathy

The 23 patients with ungradable images were automatically referred, and recommended for further in-person examination. Fifteen of them consulted ophthalmologists during 1 month after BCDRT examination,. The finding of their eye diseases was as follows: moderate to severe cataract in 8 patients, corneal scar in 3 patients, glaucoma with small pupil size in 1 patient, strabismus in 1 patient, nystagmus in 1 patient and head tremor in 1 patient. Urgent referral was recommended for patients with suspected pathology such as central retinal vascular occlusion, exudative age-related macular degeneration or glaucoma based on the appearance of the macula and optic discs. In the present study, 3 of the urgent referrals were for findings suggestive of central retinal vascular occlusion. Other pathologies found included 2 eyes with disc appearance suggestive of glaucoma.

## Discussion

The American Telemedicine Association (ATA) suggested that telehealth programs for DR should demonstrate an ability to compare favorably with ETDRS film photography as reflected in kappa values for agreement of diagnosing levels of retinopathy and macular edema [6]. However, we always found it difficult to conduct ETDRS film photography in Chinese diabetic residents, mainly because of limited ocular health care resources in Chinese communities. For this reason, we used the Chinese traditional DR screening method (in-person funduscopic examination after pupil dilation) instead, in the validation study. The high kappa value calculated herein demonstrated that our telehealth system was as valid as the traditional method for DR screening. These results are similar to that observed in former studies: there was an agreement of 85% to 94% between DR stages graded by both ophthalmologists by direct examination and by inspecting the digital images [10,20]. In a previous epidemiology study of 795 diabetic residents in the same community in 2007, we found that the prevalence rates of DR and PDR were 27.09% and 1.13%, respectively [4]. The proportions of DR and PDR (24.42% and 2.12%) revealed in this study were similar, and the small difference was plausible because of different study design, population and period. Thus, we regard BCDRT as a reliable and valid system for DR screening in local diabetic residents.

One characteristic of our DR telehealth system is that the internet-transmitted data encompass not only the digital retinographs but also visual acuity, and the latter was seldom analyzed in other DR telemedicine systems based on our MEDLINE literature search. In comparisons to other DR telemedicine systems [2,6,9,10], the BCDRT tried a simplified protocol that just acquires two 45-degree digital retinographs per eye without pupil dilation via a non-mydriatic funduscopic camera in order to reduce the screening time required and achieve greater patient acceptability. However, we did have concern that the sensitivity of detecting macular edema may decrease. For this reason, we added the visual acuity information into the digital data of BCDRT, and hoped this additional inexpensive measurement tool would trigger referral to macular edema with this system. According to the data in our report, if the diagnoses from ophthalmologists are assumed to be the "gold standard," the sensitivity and specificity of BCDRT for detecting macular edema are 88% (22/25) and 94% (29/31), respectively. These results are similar to those of Kim and associates, where the sensitivity and specificity of two non-mydriatic digital fundus image assessment of diabetic macular edema, by a retinal fellow, was 0.80 and 0.93 respectively with a 35-mm fundus image assessment as the reference standard [21]. We admit that with some stereoscopic retinal imaging systems, such as the Joslin Vision Network [20], the concordance in the gradable eyes for macular edema could be as high as 100%. However, we consider the accuracy of BCDRT diagnoses of diabetic macular edema to be acceptable, and one can expect that very few cases of macular edema will be missed using BCDRT. This system is in agreement with the Guidelines for Ocular Telehealth for DR from ATA, and belongs to the "category 2" system, which is defined as the ability to distinguish patients in two categories: (1) those who have non-sight-threatening DR or (2) sight-threatening DR [6]. In addition, the BCDRT system also guarantees the low rate of unreadable photographs for DR screening (≤ 5%) recommended by the British Diabetic Association [22]. There is good evidence that pupillary dilation reduces the proportion of patients with ungradable retinal images. For example, in the Vine Hill study, ungradable retinal images only occurred in 0.5% of the 201 screened diabetic patients under mydriasis [2]. However, pupillary dilation requires a higher level of medical supervision in many settings, and places some constraints on patients in performing certain visual tasks until the pupillary dilation dissipates [8]. The proportions of ungradable retinal images differ between DR telehealth screening systems. In the study by Ahmed and associates [20], 35% of the total images were judged inadequate to be graded fully, and the patients with ungradable eyes were significantly older than those with gradable eyes. Scanlon and associates^19 ^have previously shown that for patients 70 years and older, approximately 25% of non-mydriatic images are unreadable. A total of 74.5% (126/169) of ungradable eyes had early to obvious central cataract, and 10 eyes (6%) had a corneal scar [19]. In the present study, the proportion of media opacity in ungradable images is lower: 73.3% (11/15) of the 15 patients with ungradable images, and who were later referred to in-person examination, were diagnosed with moderate to severe cataract or corneal scar. Furthermore, we seldom encountered small pupil size and poor focus or poor patient fixation cases. Therefore, the ungradable rate in the present study (4.88%) seems to be much less than Scanlon's report. In the study by Gomez-Ulla and associates, the ungradable rate (5.2%) was similar to our result [10]. Cavallerano and associates demonstrated an even lower ungradable rate of 1.3% [23]. In the OPHDIAT study, the rate of non-gradable photographs dropped from 12.2% to 8% between September 2004 and December 2006, because technicians received additional training in an effort to improve the quality of future images [9]. Similarly, we hope the rate of non-gradable photographs in BCDRT will decrease as the PCPs have increasingly more experience with non-mydriatic image capturing.

As is well understood, the common barriers to high annual DR screening rates in most underdeveloped countries are the insufficiencies in ophthalmic equipment and experienced ophthalmologists, particularly in rural areas where access to medical resources is hindered by geographic distance. It is believed that telehealth systems help to achieve a significant increase in regular DR surveillance in underdeveloped countries, based on two characteristics inherent in these systems: (1) interactions between patients and doctors are not limited by geographic distance, (2) a well-established telehealth system facilitates centralized DR screening [9]. For example, at least 40 participants can be screened during half a day with BCDRT, which helps to achieve the maximum effects offered by medical resources. To overcome the aforementioned barriers to DR telemedicine system implementation in underdeveloped countries, we initially introduced the BCDRT system to the general residents via the mass media. As a result, although not specially queried, most of the 471 diabetic residents in this study mentioned that they had heard about BCDRT before they came to participate. The cost of necessary accessories, including a digital funduscopic camera and essential software, will impact wide-spread implementation of a DR telehealth system in some regions with limited resources. Most commercial telemedicine systems in developed countries are proprietary in that the end users (primary care centers) must use licensed software built into the imaging system for storage and transmission of images [8]. Because we did not have an adequate budget for purchase and maintenance of specific software packages after expenditures for a non-mydriatic funduscopic camera, we finally used commercially available software to construct our DR telehealth system instead, and considered it appropriate for primary care settings that serve indigent residents in underdeveloped countries. Wei and his collaborators have described some other deficiencies with the proprietary systems, including poor support for telemedicine workflow, a dependence on local area networks or dedicated computer network connections and, hence, a lack of true internet scalability and, lastly, a lack of interoperability between components from different vendors [24]. Massin and associates suggested that DR telemedicine screening centers should ideally be located in areas with a high rate of poverty and a low number of ophthalmologists [9]. Our experience with BCDRT implementation may help construction of efficient DR telehealth systems in a majority of communities and villages in underdeveloped countries, which meets the criterion of Massin's "ideal location".

A shortcoming of our study should be mentioned. First, because of limited resources, we were not able to include detailed blood laboratory studies. Second, because patients identified as normal by BCDRT were not referred to in-person examination, it is impossible to verify that the patients with normal fundus photographs indeed do not have DR on in-person examination. Therefore, we were not able to calculate additional important statistics such as sensitivity and specificity for detecting DR, because of lack of data from patients whose digital images were graded as normal. Third, the sample size is small, which leads to a high mean age and a single diabetes mellitus type in the patients studied. However, the number of participants in the BCDRT will no doubt increase with time, due to satisfactory patient preferences at the next screening (96.82%). Thus, it is promising to anticipate larger scale studies that better represent the general diabetic population, in the future.

## Conclusions

The first ocular telehealth system for DR in China - BCDRT was a reliable and valid system for DR screening. The advantages of the BCDRT are ease of use (minimal training required), convenience (located in PCP's office), and the ability to detect DR at a level requiring referral to an ophthalmologist. In the present study, we found that web technology, open source components and commercially available software can be used to build a usable, scalable, and robust telemedicine platform (BCDRT) for DR. Such an approach to software development may help to lower the cost of telemedicine systems, promote interoperability, and eventually facilitate the adoption of this new method of DR screening. Thus, the BCDRT is more appropriate to the Chinese primary care settings that serve indigent patients. With this system, we detected 115 DR out of 471 diabetic residents in 2009, so the proportion of total DR was 24.42%. BCDRT offers the potential to increase DR annual screening rates in local residents.

## List of abbreviations

ATA: American Telemedicine Association; BCDRT: the Beixinjing Community Diabetic Retinopathy Telehealth system; DR: Diabetic Retinopathy; ETDRS: Early Treatment Diabetic Retinopathy Study; NPDR: Non-Proliferative Diabetic Retinopathy; PCPs: Primary Care Physicians; PDR: Proliferative Diabetic Retinopathy.

## Competing interests

The authors declare that they have no competing interests.

## Authors' contributions

JP participated in the design of the study, contributed to the discussion and edited the manuscript. HZ participated in the design of the study, collected research data, contributed to the discussion and wrote the manuscript. WW participated in the design of the study, and collected research data. JF participated in the research data collection. BS participated in the research data collection. XB participated in the research data collection. XX contributed to the discussion and edited the manuscript. ZX contributed to the discussion and edited the manuscript. All authors read and approved the final manuscript.

## Pre-publication history

The pre-publication history for this paper can be accessed here:

http://www.biomedcentral.com/1472-6963/11/250/prepub
